# Comparison of Glenoid Dimensions Between 3D Computed Tomography and 3D Printing

**DOI:** 10.7759/cureus.53133

**Published:** 2024-01-28

**Authors:** Christos Yiannakopoulos, Iakovos Vlastos, Christos Koutserimpas, Elina Gianzina, Spilios Dellis, Georgios Kalinterakis

**Affiliations:** 1 Orthopaedics, IASO General Hospital, Athens, GRC; 2 School of Physical Education & Sports Science, National & Kapodistrian University of Athens, Athens, GRC; 3 Orthopaedics and Traumatology, IASO General Hospital, Athens, GRC

**Keywords:** scapula, glenoid dimensions, computed tomography, 3d printing, 3d imaging

## Abstract

Introduction: Glenoid dimensions can be measured in vivo with various imaging methods including two-dimensional (2D) and three-dimensional computed tomography (CT) and magnetic resonance imaging scans. Printing of three-dimensional (3D) models of the glenoid using imaging data is feasible and can be used to better understand skeletal trauma and complex skeletal deformations such as glenoid bone loss in patients with shoulder instability. The purpose of this study was to compare measurements of glenoid dimensions on 3D CT scan reconstructed models and 3D printed models of the glenoid.

Methods: CT scans from 62 young, male adults acquired for non-trauma-related causes were evaluated. Following volume rendering, a stereolithography model of each scapula was constructed and a 3D model was printed. Additionally, 3D CT models of each glenoid were reconstructed using dedicated software. Measurements of the maximum glenoid height and width were performed on both the 3D printed and the 3D reconstructed models. To assess intra- and interrater reliability, measurements of 15 glenoids were repeated by two observers after three weeks. The measurements of the 3D printed and 3D reconstructed models were compared.

Results: Inter- and intra-rater reliability was excellent or perfect. Analysis of height and width values demonstrated a strong correlation of 0.91 and 0.89 respectively (p<0.001) for both the 3D printed models and the 3D reconstructed models. There was a strong correlation between the height and width, but no significant difference between the glenoid width and height in both models. There was no statistical significance between height and width when measurements on the two models were examined (p=0.12 and 0.23 respectively).

Conclusion: 3D printed glenoid models can be used to evaluate the glenoid dimensions, width, and height, as they provide similar accuracy with 3D reconstructed models as provided from CT scan data.

## Introduction

The glenoid fossa or cavity is a shallow, convex depression of the lateral angle of the scapula, which articulates with the convex head of the humerus to form the glenohumeral (GH) joint. The GH joint is a synovial ball-and-socket diarthrodial joint that is very mobile and inherently unstable and its biomechanical stability relies on the integrity of the GH ligaments. The size of the glenoid is significantly smaller than the diameter of the humeral head, allowing a greater range of motion but increasing the propensity for joint dislocation [1]. Shoulder dislocations represent 50 percent of all major joint dislocations and the most common direction of dislocation is anterior [2].

The morphology of the glenoid cavity, in health and disease, is variable. The glenoid is usually pear-shaped or inverted comma-shaped, although an oval-shaped glenoid is not uncommon [3]. The dimensions of the glenoid depend on ethnicity and sex [4-6]. There is a significant correlation between the patient height and glenoid size [6,7], but there are no significant side-dependent differences in the osseous anatomy of the glenoid [8,9] or the humeral head [8].

The morphology of the glenoid is significantly altered in patients with GH instability [9] and GH osteoarthritis [10]. In shoulder instability, the anterior-inferior part of the glenoid is eroded depending on the number and severity of the dislocation episodes [9], while in osteoarthritis the glenoid is eroded concentrically or eccentrically [10]. In osteoarthritis, the dimensions of the glenoid are increased because of bone erosion and the presence of osteophytes [10].

The morphology of the glenoid can be evaluated using radiographs, computed tomography (CT) scans, and magnetic resonance imaging (MRI). CT scans are used to quantify the dimensions of the glenoid using multiplanar reformation (MPR) or 3D reformatted images of the glenoid [11-13]. Quantification of the glenoid morphology is essential in shoulder instability surgery because glenoid bone loss is the most critical factor for Bankart capsular repair failure [14].

3D printing technology has significantly evolved in the last years and currently, patient-specific 3D printed models are used as surgical and educational tools [15,16]. 3D printing of the glenoid based on pre-operative CT-derived data has been used for the preoperative planning of shoulder arthroplasty [17] or for the evaluation of glenoid morphology in osteoarthritis [18,19].

The purpose of the current study was to compare the accuracy of the measurement of glenoid dimensions comparing 3D computed tomography reconstruction models and 3D printed models in a series of patients without glenoid pathology or post-traumatic deformation.

## Materials and methods

**Figure 1:**
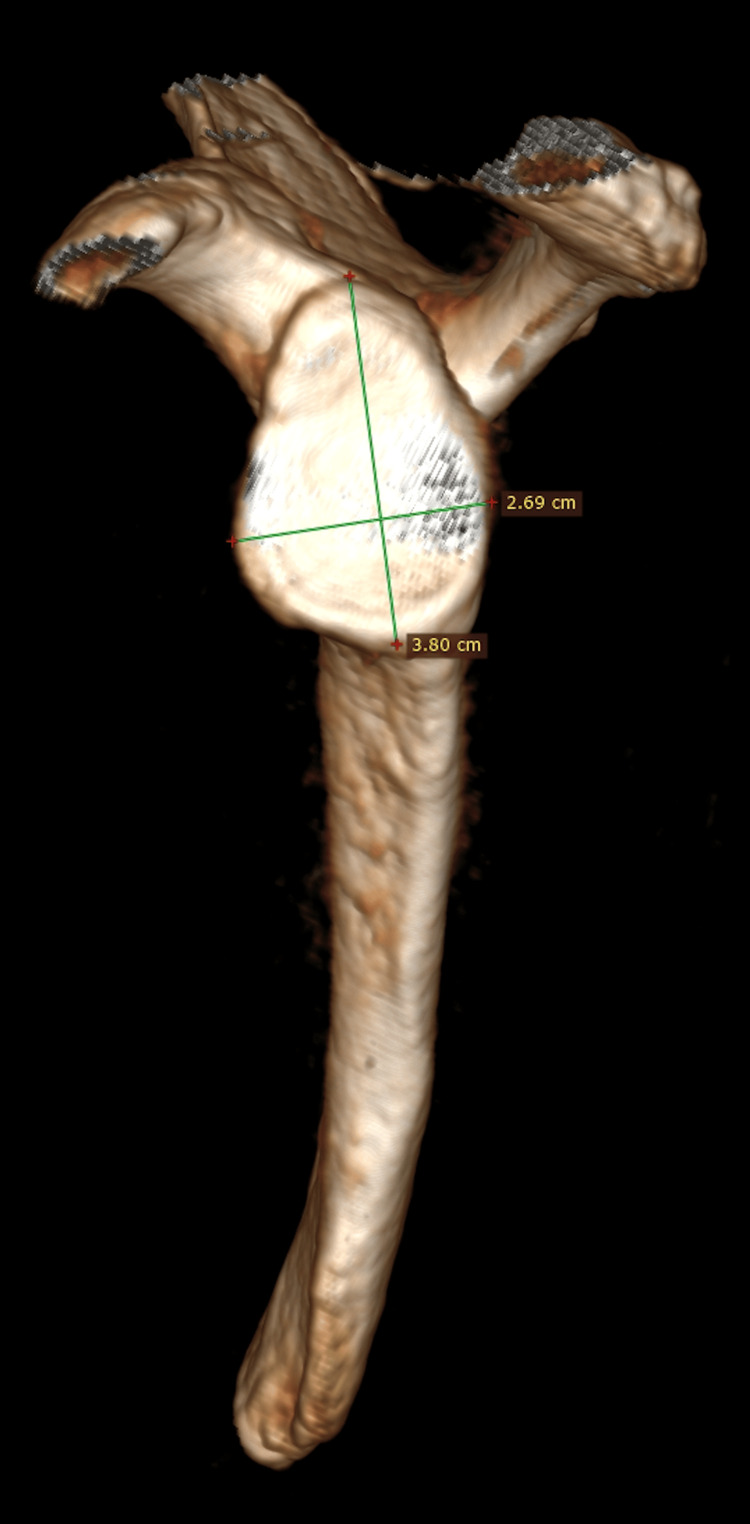
A 3D CT reconstructed model of the glenoid. A 3D CT reconstructed model of a right glenoid. Measurement of the maximum superoinferior length and the maximum anteroposterior width is possible.

We examined CT scans from 62 young, male adults aged 23 to 38 years, acquired for non-traumatic causes. The study was approved by the Institutional Scientific Committee (approval number 1145/26/09/2022). There were 42 right and 20 left shoulders. All CT scans were acquired using a standard clinical protocol composed of a helical scan (140 kVp and 200 mA) with a slice thickness ≤1 mm and interval: ≤0.625 mm. The CT scan was performed with the patient supine with his arms at the side with the shoulder in neutral rotation centered in the gantry. Span spacing was set at zero, the slices were continuous, and the gantry angle was 0o, without tilt. DICOM (Digital Imaging and Communications in Medicine) data from each CT scan were loaded to Horos, an open-source code software (FOSS) program that is distributed under the LGPL license (https://horosproject.org/). Using the 3D volume rendering function, the scapula was isolated, and a three-dimensional model of the glenoid was constructed. The maximum glenoid height was initially measured as the maximal length from the superior pole of the glenoid to the inferior pole, which did not always coincide with the 12 to 6 o’clock position and was occasionally disconcerting (Figure 1).

**Figure 2:**
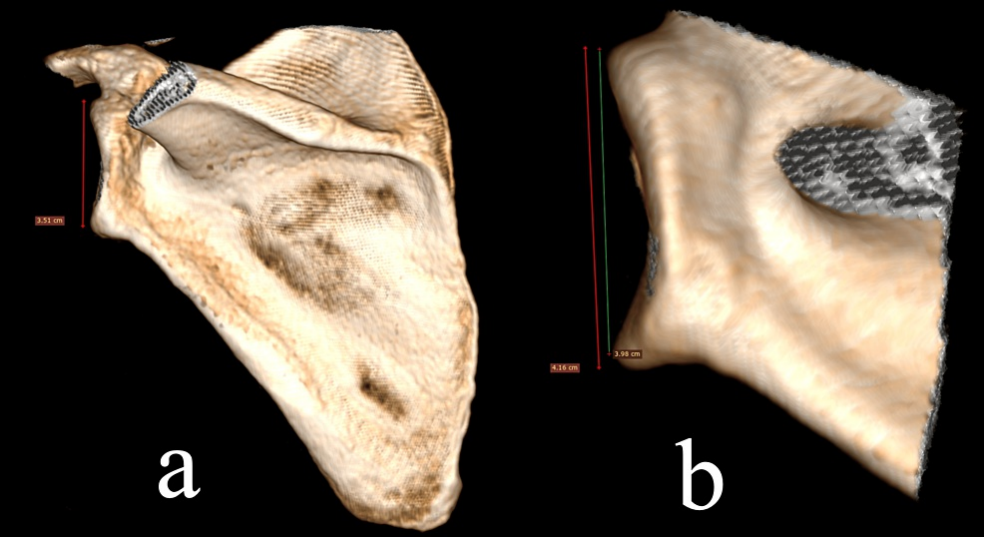
Measurement of the maximum glenoid height. We chose to measure the maximum superoinferior length of the glenoid in a true anteroposterior projection of the scapula (a) because measuring the glenoid length on the true lateral view may lead to overestimation to difficulty in discriminating the upper and lower limits of the glenoid (b).  The red line in (b) represents the measurement of the glenoid height as it would be performed in the true lateral view, whereas the green line is the actual method of measurement used in the current paper. Using this method, the articular surface of the glenoid is more accurately measured.

Thus, the glenoid height was measured by rotating the 3D model until the glenoid surface was seen in an anteroposterior direction (Figure 2a). When the height of the glenoid is measured in a lateral 3D reconstructed model, the effect of the glenoid superior-inferior inclination is not considered (Figure 2b) and this may lead to inaccuracy. 

**Figure 3:**
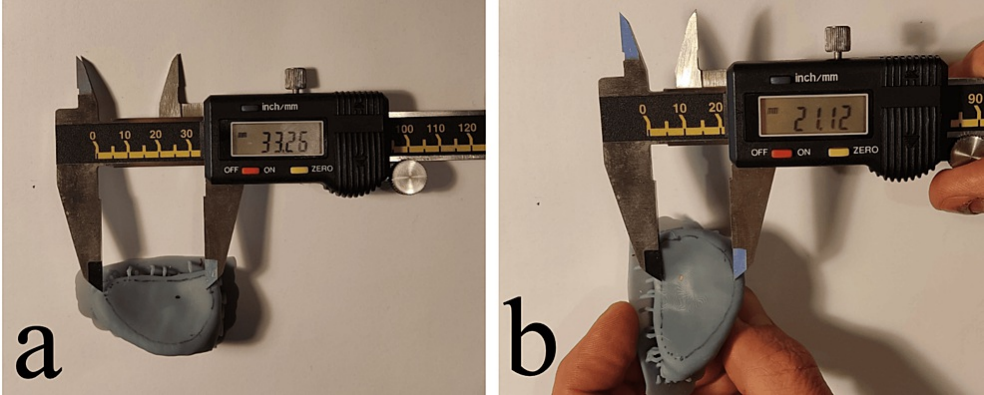
Measurement of the glenoid dimensions on a #D printed model. Measurement of the glenoid length (a) and width (b) using a digital caliper.

The maximum width of the glenoid was recorded in an orthogonal orientation to the maximum glenoid height (Figure 1). For the construction of the 3D printed models, DICOM data were loaded using the 3D slicer (https://www.slicer.org) and meshmixer (https://meshmixer.com) software, a stereolithography model was constructed and printed on a desktop 3D printer (Anycubic Photon M3 Plusresin printer) with printing accuracy 6K (5.760 x 3.600 pixels). The glenoid height and width were measured using a high-precision digital ruler (Figure 3). 

All data were recorded and presented as mean values and standard deviation. Intra-class correlations, two-way random effects model, ICC (2.1), were used to measure the inter- and intra-rater reliability for the quantitative measurements of the glenoid width and height. Values less than 0.5 are indicative of poor reliability, values between 0.5 and 0.75 indicate moderate reliability, values between 0.75 and 0.90 indicate good reliability, and values greater than 0.90 indicate excellent reliability. Pearson correlation coefficients (r) were calculated for both glenoid height and width for each method. The difference of height and width between the two models (3D printed and 3D reconstructed) was evaluated using a paired t-test. Statistical analysis was conducted with IBM SPSS Statistics for Windows, Version 28 (Released 2021; IBM Corp., Armonk, New York, United States) and the level of statistical significance, p, was set at 0.05.

## Results

Intra-rater reliability was found to be good to excellent, with an ICC of 0.876 for glenoid width and 0.92 for height for the 3D printed and 0.93 for glenoid width and 0.90 for height respectively for the 3D reconstructed models. Inter-rater reliability testing also showed high levels of agreement, with ICC values of 0.91 and 0.969 for width and height for the 3D printed and 0.929 for the glenoid width and 0.915 for the glenoid height for the 3D reconstructed models, respectively. 

**Figure 4:**
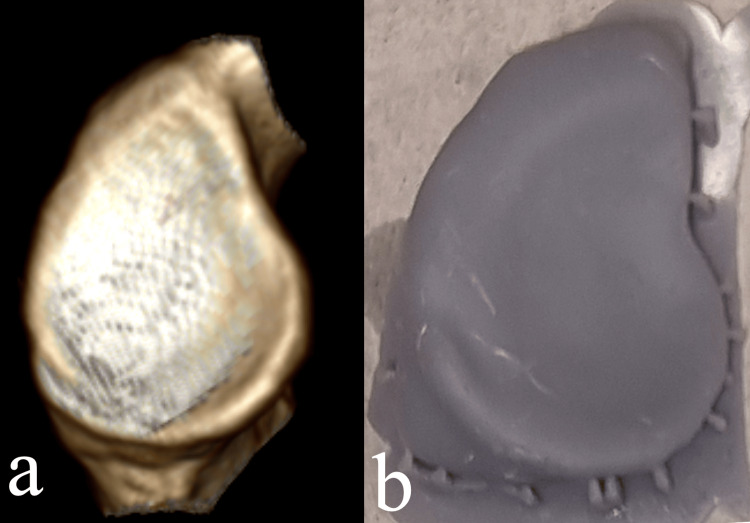
Visual comparison of the 3D CT reconstructed glenoid models and the 3D printed models. On visual inspection, there is no difference in the morphology of the 3D CT reconstructed glenoid models (a) and the 3D printed models (b).

The mean height and width in the 3D CT models were 36.9 ± 3.2 mm (range 28.6-40.2 mm) and 24.4 ± 2.67 mm (range 23.1-30.8 mm) respectively and in the 3D printed models 36.1 ± 2.2 mm (range 28.1-40.3 mm) and 25.2 ± 2.86 mm (range 23.5-30.4 mm) respectively. The comparison for height and width revealed a statistically non-significant difference (p=0.21 and 0.54 respectively). Visually, the 3D CT models and the printed glenoids were similar (Figure 4).

Analysis of the height and width values demonstrated a strong correlation of 0.91 and 0.89 respectively (p<0.001) for both the 3D printed models and the 3D reconstructed models. There was no statistical significance between the height and width when measurements on the two models were examined (p=0.12 and 0.23, respectively).

## Discussion

The primary aim of the study was to compare the accuracy of the measurement of glenoid dimensions comparing 3D computed tomography reconstruction models and 3D printed models in a series of patients without glenoid pathology or post-traumatic deformation. Our study showed that there is a good correlation between glenoid height and width as measured on 3D CT reconstructed and 3D printed glenoid models. The measurements in both models showed excellent inter- and intra-rater reliability. 

We measured the glenoid length in the anteroposterior projection to avoid inaccuracy in identifying the superior and inferior borders of the glenoid as we noticed to occur in the lateral projection. A similar method to measure the length of the glenoid has been used on 2D CT scans [20]. 3D CT is accurate in predicting the true anatomy of the glenoid [21] and can accurately predict native glenoid width [7]. In the study by Kwon et al., various morphological parameters were measured both on 3D CT scans and on cadaveric scapulae [21]. The glenoid surface width and length from the 3D CT images were within 1.8 ±1.2 mm and 1.4 ±1.1 mm, respectively, of those from the glenoid cadaveric specimens [21]. Zhou et al. compared several glenoid morphologic parameters using cadaveric scapulae and 3D CT reconstructed models [22]. They showed no significant differences between manual and CT measurements. 3D CT is superior to 2D MRI in measuring the glenoid height and subsequently calculating the glenoid width [23]. In our study, we have shown that 3D printed glenoid models can be used to evaluate the glenoid dimensions. 

Inter- and intra-rater reliability in our study was excellent or perfect because the raters are very experienced in the assessment of shoulder CT in normal and pathological conditions. In the study by Giles et al. [7] intra- and interrater reliabilities were good to excellent for height and width, with intraclass correlation coefficients of 0.765 to 0.99 [7]. Kubicka et al. evaluated the reliability of glenoid dimension measurements using 2D-CT and 3D-CT scans [11]. They showed that for both observers participating in the study, almost all glenoid parameters differed significantly between 2D and 3D measurement methods [11]. Al Najjar et al. compared 3D CT models and 3D printed models and found significant inter-rater reliability with intraclass correlation ranging between 0.94 and 0.99 [18].

3D printed models provide an accurate measurement of in vivo bones. 3D printed models of the glenoid based on CT data have been used in patients with glenohumeral osteoarthritis prior to shoulder replacement [17,19]. Printed models facilitate understanding of the deformed glenoid morphology and can be used pre- and intraoperatively [17]. Shah et al. compared 3D printed scapula models and 3D CT scan models in a series of 20 patients with shoulder osteoarthritis [19]. They found a significant discrepancy in the measurement of the glenoid inclination and version between the 2 models. Al Najjar et al. compared 3D CT models and 3D printed models using a desktop printer in 32 patients with shoulder osteoarthritis [18]. They compared the glenoid version, the glenoid maximum height and width, and the maximum acromion antero-posterior length between the two models. They found no statistically significant difference in the maximum width measurements contrary to the mean difference for the glenoid height which was 3.67±12.04 mm. Willemsen et al. in a cadaveric study used 3D printing to produce an anatomy-specific titanium implant to restore a glenoid defect [24].

Our study has several limitations. We collected CT scans only from male patients and thus the possible effect of the gender on the glenoid dimensions was not assessed, although it can be assumed that it will be negligible. Our database did not include glenoids from patients with shoulder instability and bone defects, and this needs to be further evaluated. We used a desktop 3D printer to produce the printed models. More expensive printers with higher accuracy will improve the quality of printing results without reducing the accuracy of the glenoid dimensions. It has been shown, though, that desktop 3D printers are accurate in printing skeletal models for anatomy teaching, preoperative planning, and intraoperative orientation [16]. Finally, the results of the study are valid only for the software used for 3D printing and the creation of 3D CT models.

## Conclusions

Measurement of the glenoid length and width on 3D printed glenoid models provides similar results with 3D reconstructed models from CT scans and can be used as a tool for studying normal glenoid anatomy. Evaluation of the 3D printing method in patients with glenoid deformity due to chronic shoulder instability or glenohumeral osteoarthritis remains to be performed.
